# Supplementary material for: Disruption of the NF-κB/IL-8 Signaling Axis by Sulconazole Inhibits Human Breast Cancer Stem Cell Formation
Source: Cells. 2019 Aug 30;8(9):1007. doi: 10.3390/cells8091007 (PMC6770215; doi:10.3390/cells8091007)
Supplement: Supplementary file 1 [file cells-08-01007-s001.pdf]

Table S1. Specific primer sequence of Real-time RT-qPCR

Genes	Primers
**CD44**	Forward: 5′-AGAAGGTGTGGGCAGAAGAA-3′<br>Reverse: 5′-AAATGCACCATTTCCTGAGA-3′
**Nanog**	Forward: 5′-ATGCCTCACACGGAGACTGT-3′<br>Reverse: 5′-AAGTGGGTTGTTTGCCTTTG-3′
**C-myc**	Forward: 5′-AATGAAAAGGCCCCCAAGGTAGTTATCC-3′<br>Reverse : 5′-AGCAAAACCCGGAGGAGT-3′
**β-actin**	Forward: 5′-TGTTACCAACTGGGACGACA-3′<br>Reverse : 5′-GGGGTGTTGAAGGTCTCAAA-3′
